# Current Insights into Surgery for Intramedullary Spinal Cord Metastases: A Literature Review

**DOI:** 10.1155/2011/989506

**Published:** 2011-05-26

**Authors:** Ondrej Kalita

**Affiliations:** Neurosurgical Department, University Hospital and Medical Faculty of Palacky University Olomouc, I.P. Pavlova 6, 77520 Olomouc, Czech Republic

## Abstract

Intramedullary spinal cord metastasis (ISCM) is the rarest type of CNS involvement by systemic malignant tumours. Optimal management of patients with ISCMs remains ambiguous. Based on two cases reported from our department, we focused on the strategy for intramedullary spinal cord metastases surgery.

## 1. Introduction

Intramedullary spinal cord metastases (ISCMs) are a rare complication of malignancy and have been identified in 0.9–2.1% of all neoplasm autopsies. ISCMs are often asymptomatic and clinically affect only 0.1–0.4% of all cancer patients. One-quarter of ISCM patients have leptomeningeal carcinomatosis, and one-third have concomitant brain metastases [1–5]. Additionally, ISCMs commonly create solitary lesions, constitute 8.5% of all central nervous system metastases, and comprise 4% to 9% of all spinal cord tumours. On the contrary, intracerebral metastases occur in 20–50% of all cancer patients, and multiple lesions develop in 30–50%. Despite the existence of tissue perfusion differences, the distribution of metastases in the brain and spinal cord has not been satisfactorily explained.

Optimal management of patients with ISCMs is difficult due to the wide variety of clinical situations and the lack of controlled studies on the results of different therapeutic options. Therapeutic options for ISCMs include microsurgical excision; radiotherapy, currently mainly stereotactic radiotherapy; chemotherapy; palliative therapy, particularly steroids. Many authors have advocated radiation therapy, preferably for radiosensitive metastases such as small cell carcinoma, breast cancer, or lymphoma [1, 4, 6, 7]. From our point of view, surgery could be considered an optimal therapeutic approach for ISCMs, even accompanied by an acceptable postoperative functional outcome.

## 2. Materials and Methods

In the recent ten years, there has been substantial progress in oncotherapy, microsurgery, and MRI availability. We researched ISCM cases in Pubmed during this period, including retrospective analyses and meta-analyses. This resulted in limited bias due to the inclusion of ISCM surgery cases from the previous period. Both spinal extradural and primary brain metastases were beyond the scope of this paper and were excluded. Finally, cases of ISCMs managed by surgery were selected.

## 3. Results and Discussion

Eleven papers concerned with ISCM surgery have been published in the last ten years. Except for one review article, only case reports were found. However, two more abundant studies with 13 and 19 patients were also discovered. To sum up, a total of fifty-four patients underwent surgery. Laminectomy or osteoplastic laminotomy with tumour excision was performed in all but one. In this single case, only palliative decompressive laminectomy was done [8]. None of these developed new neurological deficits, and partial neurological improvement was achieved [1, 2, 4]. 

In the literature, tumour removal was identified as complete in 47 patients and incomplete in 5 patients, and surgical biopsy was done in 2 patients [1–10].

### 3.1. Incidence and Pathophysiology

Lung metastases, which account for about half of the ISCMs, were by far the most common source. The incidences of the various tumour primaries were lung (especially small cell carcinoma) 29–54%, breast 11–14%, kidney 6–9%, colorectal 3–5%, melanoma 6–9%, lymphoma 4%, thyroid 2%, ovarian 1%, and approximately 3% were categorized as secondary to an unknown primary [11].

**Figure 1:**
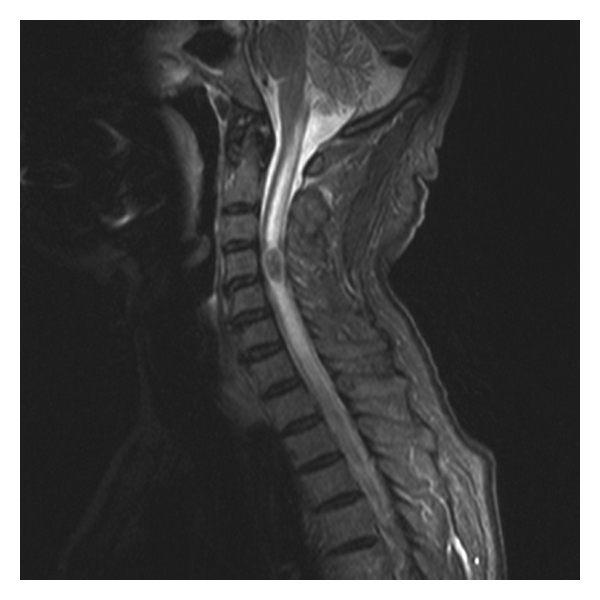
Intramedullary metastasis of colorectal carcinoma.

**Figure 2:**
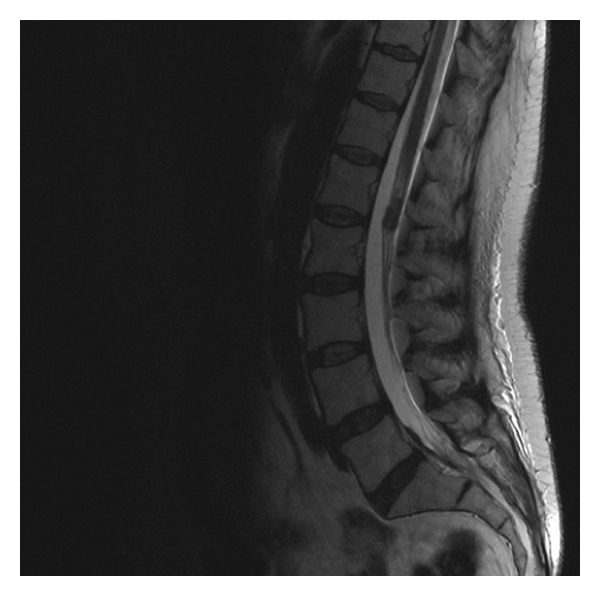
Cauda equine infiltration by breast carcinoma.

**Figure 3:**
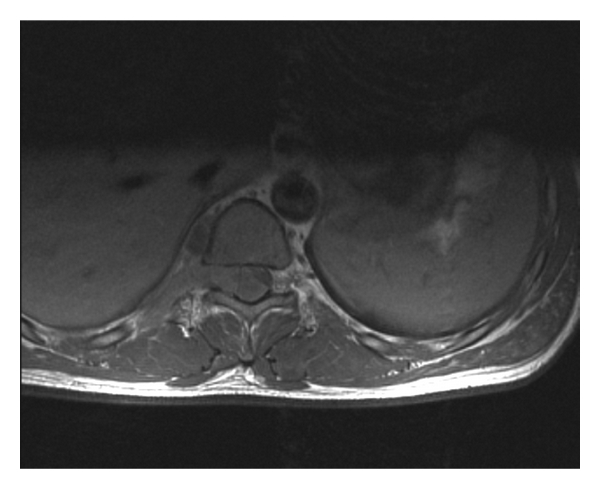
Direct intraspinal cancer invasion along nerve root.

ISCMs are mainly disseminated by the arterial route (Figure 1). Meningeal carcinomatosis occurs by CSF seeding. Tumour cells infiltrate the Virchow-Robin spaces of vessels, penetrating the spinal cord and pial membrane and invading the spinal cord parenchyma (Figure 2). A third mechanism is the direct invasion from contiguous structures. Spread through the vertebral venous plexus (Batson's plexus) during Valsalva manoeuvre enabling retrograde blood flow to the spinal cord is also possible. ISCMs concurred with meningeal carcinomatosis in 15–55% of cases. Finally, direct invasion from contiguous structures is also conceded. Although the dura protects the cord from invasion by malignant tumours, metastasis expansion from the spinal extradural space or nerve roots through the dura and into the cord has been suggested (Figure 3) [1, 12].

The above-mentioned discrepancy between the frequencies of brain and intramedullary metastases would be partly explained as follows. One-third of the cardiac output through the large vessels under high pressure directs to the brain, while the spinal cord receives arterial supply from small, low-pressure, and convoluted vessels. The medullary arteries branch off the aorta at right angles, while the cerebral arteries are almost a direct extension of the aorta, thus favouring embolic seeding [9].

### 3.2. Symptoms

Due to the advances in imaging and therapeutic modalities prolonging the survival of cancer patients, the probability of discovering ISCMs increases. 

ISCMs may be considered as an infrequent finding at advanced stage of disease, usually accompanying rapid progression of systemic cancer. But in much rarer cases, ISCMs are the initial presentation of malignancy. This occurred in 22.5% to 39% of ISCM cases [1, 4, 7].

Prompt diagnosis is important for treatment rationale. However, exact diagnosis of ISCMs may be difficult despite a positive history of malignancy, because clinical findings do not help to distinguish ISCMs from other lesions such as epidural metastasis, paraneoplastic necrotizing myelopathy, radiation myelopathy, or from a coincidence of nutritional, demyelinative, inflammatory, or vascular myelopathies [1, 7].

The published median intervals between the onset of ISCM-related neurological symptoms and the final diagnosis varied from 1 week to 2 weeks to 7 weeks to 9 weeks, with the greatest range being 17 months [1, 2, 4, 6, 7]. 

At diagnosis, about 20% of patients are ambulatory independent, 40% are ambulatory with assistance, and the remaining 40% are nonambulatory. Nevertheless, one author somberly reported that three-fourths of patients developed a complete neurological deficit during less than one month from the onset of neurological symptoms [1, 2]. The described rapid symptom progression is elicited by the limited resistance of spinal cord tissue and vascular structures involved by an increasing tumour size and perifocal oedema. ISCMs predominantly affect elderly patients who commonly have serious comorbidities and, thus, myelopathy presents more acutely in them.

The range, pattern, and deterioration of the neurological status are based on ISCM location and volume. Clinically, ISCMs are manifested by pain, weakness, sensory loss, and incontinence. Brown-Sequard syndrome or complete spinal cord transection is nearly equally present. Pain is usually followed by sensory and sphincter disturbance [1, 2, 11].

Weakness was present in 91% of patients at diagnosis of ISCMs. Sensory loss (79%), sphincter dysfunction (60%), back pain (38%), and radicular pain (24%) were also common. Brown-Sequard syndrome or pseudo-Brown-Sequard syndrome was seen in 23–45% of cases. Asymptomatic cases have also been described (1%) [1, 2, 7, 10, 11].

### 3.3. Diagnosis

Currently, spinal magnetic resonance imaging (MRI) is applied routinely for the diagnosis of ISCMs. Before the era of MRI, only 5% were recognized before death. Other imaging techniques (CT, PET/CT, and angiography) are of marginal significance. Single ISCM is most commonly seen (94%) although multiple ISCMs (6%) are also encountered [1, 7]. 

Due to the general infrequency of ISCMs and the time elapsed from the first cancer attack, tumour duplicity, that is primary intraspinal neoplasm, would be the preferred diagnosis. ISCMs are typically characterized by rapid progression of symptoms in contrast to the relatively slow growth of primary intramedullary tumours (gliomas, ependymomas, etc.). 

At the time of diagnosis, 55% of ISCM patients had systemic metastases, and an additional 41% had brain metastases. The other types reported included bone (24%), leptomeningeal (17%), pulmonary (13%), lymph node (12%), hepatic (8%), adrenal (2%), splenic, sacral, and rib metastases.

Cytological investigation of CSF is usually negative. Retrospectively, malignant cells in CSF were confirmed only in 11 patients. Nearly, in all ISCM patients (95%), CSF protein level was abnormally elevated. In older studies, one-half to two-thirds of meningeal carcinomatosis cases had malignant cells in CSF analysis. Thus, lumbar puncture and CSF analysis have limited significance for the diagnosis of ISCMs [1, 2, 4, 7].

### 3.4. Surgery and Survival

Currently, surgical approach is more precise and less invasive and thus allows spinal cord tumour excision with an acceptable morbidity rate. The purpose is decompression of functional neural tissue and histological confirmation of the tumour. Some authors documented that complete neurological deficit developed in 75% of ISCM patients within approximately one month. Given this result, surgical resection can be performed in these selected cases [1, 2].

Advanced technologies such as MR/CT navigation or ultrasound for tumour location, cavitron ultrasonic surgical aspirator and laser for dissection, and intraoperative monitoring of somatosensory evoked potentials have allowed limited myelotomy and a relatively safe tumour removal. Radicality also depends on tumour histology. Patients with adenocarcinoma had longer survival rates than those with poorly differentiated carcinomas. Poorly differentiated carcinomas and sarcomas are difficult to be managed by radical surgery due to the absence of a clear cleavage plane. In these cases, some authors recommend biopsy, decompressive laminectomy, and adjuvant therapy [2, 7, 13].

Many reports favoured radiotherapy (RT). However, given the rarity of ISCMs, no controlled studies comparing surgery and RT were undertaken.

The median survival of patients with ISCMs depends on several conditions. The survival was influenced by the preoperative neurological status, nature of the primary cancer, and presence of systemic and/or CNS metastases, but the differences were mostly without statistical significance. Nevertheless, surgery statistically prolonged survival more than doubly (7.4 versus 2.6 months). According to other authors, the median survival extends beyond 9.4 months when patients undergo surgery versus 5 months when conservative treatment is performed. Regarding primary cancer, the median survivals are 5.5 months and 1.0 month for breast cancer and lung cancer, respectively. Most patients succumbed to progression of the primary cancer [2, 8, 9].

The neurological status improved in 58% (11/19) of operated patients. On the contrary, ISCM patients with brain metastases have a life expectancy of about 3 to 4 months from the time of diagnosis [14].

### 3.5. Adjuvant Therapy

Focal radiation is used as an adjunct treatment for residual disease within the resection cavity. Postoperatively, RT (30 Gy) is used in the resection site. 

The majority of ISCMs published were only treated with radiotherapy or chemotherapy. The aim of RT is to arrest tumor growth and prevent further neurological deficit. RT is considered for radiosensitive carcinomas such as small cell lung carcinoma, breast carcinoma, or lymphoma. The reported six-month survival rate of the cases treated with radiation therapy is less than 20%. The response to RT is minimal if paraplegia supervenes [14, 15]. 

Moreover, RT is controversial in many cases. The risk of radiation myelitis can occur after significant radiation exposure. The radiation tolerance of the spinal cord alone is substantially limited, and knowledge of the safe amount of radiation delivered to the spinal cord involved by metastasis is lacking [16]. Fractionated radiotherapy is effective particularly when the patients are not neurologically compromised. Currently, much more targeted RT such as stereotactic radiotherapy is recommended. Some authors even advise to irradiate the entire spinal cord, but with unknown bone marrow toxicity and a questionable improvement in the outcome.

Most of these patients underwent chemotherapy and biological therapy during the first cancer attack, but these are not applicable in ISCM management. These modalities do not pass through the blood-spinal cord barrier. Chemotherapy has no further effect on survival [7, 17–19].

Another therapeutic modality is steroid therapy. Patients with rapidly progressive symptoms of cord compression and a high risk of rapid deterioration are suitable for treatment with high-dose steroids. This may decrease the pain and cause transient improvement in neurological conditions. Steroids suppress perifocal oedema and normalize the blood-spinal cord barrier, which decreases tumour size but does not influence survival. Steroid therapy provides additional time for the diagnostic process as well as controls or minimizes the oedema after surgery or RT [2].

## 4. Conclusion

Diagnosis should be made as early as possible and surgical resection should be considered as the primary treatment whenever feasible, particularly in the case of rapidly progressive neurological deficits.

Our strategy involves aggressive surgery in selected ISCM patients, particularly those presenting with previously undiagnosed or limited primary tumours. Before choosing surgery, the clinical condition, age of the patient, primary tumour pathology, and presence of other secondary lesions have to be assessed. Finally, the expected survival is estimated, and the rationale for the surgical procedure as well as the approach and extent of resection are obtained. An intraoperative monitoring is auxiliary method to facilitate safe and radical tumour resection. 

CNS metastases are extra-axial tumours. The neoplasm characteristics play a major role. ISCMs are often well circumscribed, cystic or with a cystic part, and encapsulated. Cystic tumours or cystic tumour components facilitate surgical extirpation because the cystic component often reaches the spinal cord surface. Therefore, the tumor can be easily removed without disruption to normal neural structures. However, gross total resection of solid tumour with preservation of neurological functions is possible. In the selected patients, the quality of life can be improved. Surgery should not be undertaken in cases of complete paraplegia. 

The author has had two published experiences of ISCMs. Firstly, there was a patient with solitary ISCM as initial sign of colorectal carcinoma. Due to fast neurological deterioration the patient underwent ISCM surgery followed solution of primary tumour and extraneuronal metastases. Secondly, there was a patient with history of breath carcinoma. She has been already treated for extraneuronal metastases several years. A neurological deficit evolved, and solitary ISM was revealed. For relatively good prognosis of this carcinoma, the patient underwent surgery. 

Although the number of cases is small, analysis of literature data suggests that, in some patients with ISCMs, surgical treatment is of relevance.

The median progression-free survival was 13 weeks (range 3–96 weeks), and the median survival time after surgery was 27 weeks (range 3–148 weeks). The median survival of patients with adenocarcinoma metastases was 42 weeks. The median survival was 21 weeks and 8 weeks for sarcomas and poorly differentiated carcinomas, respectively [1, 2, 7, 20].

RT is considered as an adjunct treatment after surgery.

Some authors have proposed the possibility that chemotherapy for intracerebral metastases can cross the disrupted blood-brain barrier and extrapolated this to ISCMs [17–19]. When compared, the blood-brain barrier and the blood-spinal cord barrier are not entirely identical. For example, their permeabilities are reduced by steroids.

Patients with ISCMs have a limited response to treatment and a very unfavourable prognosis. Every effort should be made for effective palliation and prevention of paraplegia. In our view, it is of essential importance to minimize the time from the first symptoms to diagnosis and to select patients who would benefit most from surgery. Ultimately, cooperation among the neurosurgeon, neuroradiotherapist, neuro-oncologist, neuropathologist, and physiotherapist is absolutely mandatory for an effective therapeutic process.
